# Delivery of Outpatient Parenteral Antimicrobial Therapy (OPAT) in an Ever-Changing National Health Service (UK): Benefits, Barriers, and Opportunities

**DOI:** 10.3390/antibiotics14050451

**Published:** 2025-04-29

**Authors:** Oyewole Christopher Durojaiye, Charlotte Fiori, Katharine Cartwright

**Affiliations:** 1 Department of Infection and Tropical Medicine, Sheffield Teaching Hospitals NHS Foundation Trust, Sheffield S10 2JF, UK; katharine.cartwright@nhs.net; 2 OPAT Unit, University Hospitals of Derby and Burton NHS Foundation Trust, Derby DE22 3NE, UK; charlotte.fiori@nhs.net

**Keywords:** benefits, National Health Service, OPAT, outpatient parenteral antimicrobial therapy, risks, UK

## Abstract

Outpatient parenteral antimicrobial therapy (OPAT) is increasingly used to manage a broad range of infections, enabling patients to receive intravenous antibiotics safely outside inpatient settings. In this review, we examine the current landscape of OPAT practice across the United Kingdom (UK), assessing its clinical, economic, and operational impact. The benefits of OPAT for patients and the National Health Service (NHS), as well as its associated risks, are discussed. Additionally, we explore the challenges hindering its broader implementation within the UK. Finally, we highlight recent innovations and emerging applications of OPAT relevant to the NHS, underscoring key considerations for its future expansion and emphasising the need for a nationally coordinated strategy to realise its full potential.

## 1. Introduction

The practice of administering intravenous (IV) antimicrobials outside inpatient settings, known as outpatient parenteral antimicrobial therapy (OPAT), was first described in the United States (US) in 1974 for the treatment of infectious exacerbation of cystic fibrosis in a paediatric population [1]. Since then, it has become a standard of care in many parts of the world for managing a wide range of infections that require prolonged courses of IV antibiotics [2,3].

In the UK, OPAT has evolved relatively slowly but is now increasingly adopted across both the National Health Service (NHS) and the private sector, as the benefits of improving patient outcomes while simultaneously reducing the burden on local healthcare systems are being recognised [4,5]. OPAT offers considerable opportunities to improve efficiency within the NHS [6]. For example, a London-based NHS trust reported estimated annual savings of £1–2 million due to reduced hospital stays through OPAT implementation [7].

Currently, the NHS faces significant challenges, including financial constraints and rising demands for healthcare services. While OPAT has strong potential to reduce healthcare costs and supports key reform initiatives within the NHS [8], its full benefits are not being realised due to patchy implementation and significant variation in practice across the country [5,9]—with no national OPAT commissioning policy currently in place.

As part of the UK government’s broader push for ‘care closer to home’ and integrated community services [8], OPAT is not merely a clinical innovation but a strategic lever in reshaping acute care delivery across the NHS. In this article, we aim to describe the current state of practice of OPAT in the UK, highlighting its benefits, potential risks, and challenges, while also exploring opportunities within the NHS.

## 2. OPAT in the UK

### 2.1. Clinical Practice

OPAT in the UK was first described in the early 1990s [10] and was initially limited to a few specialist centres. Over time, it has expanded significantly, though often in an ad hoc manner [4,5]. The growth of OPAT in the UK can be attributed to several factors, including financial pressures, an increasing focus on delivering care outside of hospital settings, greater acceptance of home-based treatment by both patients and healthcare professionals, and medical advancements such as long-acting antimicrobial agents, portable infusion devices, and improved vascular access technology [4,11]. Additionally, OPAT has been advocated as a key component of the UK government’s antimicrobial stewardship (AMS) initiative—‘Start Smart-Then Focus’ [12]—and the 5-year national action plan for antimicrobial resistance 2024 to 2029 [13].

Following expansion across the UK, OPAT services now range from well-structured, dedicated units to more informal services integrated within broader healthcare provision [4]. They are delivered in a variety of clinical settings, including outpatient clinics, ambulatory care units, primary care facilities, and non-clinical environments [4]. OPAT may be provided entirely in the community or initiated in a hospital setting and completed through a community-based service. The management of OPAT services now involves a diverse range of healthcare professionals, including acute and general physicians, advanced nurse practitioners, specialist nurses, and clinical pharmacists, rather than being solely led by infection specialists [4]. Despite the growth of OPAT, paediatric services remain limited in the UK and are primarily restricted to tertiary referral hospitals [5,14].

In the UK, OPAT is generally delivered through three models of care: the infusion centre model, where patients attend a healthcare facility (e.g., specialist OPAT clinics or outpatient clinics) daily; the visiting nurse model, where a primary care or specialist nurse administers therapy in the patient’s home; and the self-administration model, where patients and/or their caregivers are trained to administer therapy at home [4]. Increasingly, individual OPAT services in the UK offer one or a combination of these three models, depending on local constraints and logistical factors such as financial resources, workforce capacity, and the geographic spread of patients [3,15].

The criteria for selecting patients for OPAT vary across UK OPAT services [4,5]. In general, eligibility requires that individuals meet specific clinical and social criteria to ensure safety and effectiveness [4,7,10]. Clinically, patients should be medically stable, have a confirmed diagnosis requiring IV antimicrobial therapy, and exhibit a predictable treatment response. They should not require continued inpatient care and must not be at significant risk of rapid clinical deterioration. Socially, patients should have a suitable home environment, access to a telephone, and the capacity to provide informed consent and comply with treatment schedules, either independently or with support. A multidisciplinary team (MDT) assessment is typically conducted to confirm that these criteria are met prior to initiating OPAT.

A diverse range of infections has been successfully treated in UK OPAT services, including multi-drug resistant (MDR) tuberculosis [16], fungal [17], and tropical parasitic infections [18,19]. A review of a national OPAT registry maintained by the British Society for Antimicrobial Chemotherapy (BSAC) from 2015 to 2019, which contained data submitted by 57 UK OPAT services on 27,841 patient episodes, indicated that skin and soft tissue infections (SSTIs), bronchiectasis, and urinary tract infections (UTIs) were the three most common conditions treated in adult OPAT services. In paediatric OPAT services, the top three infections were ‘viral’ infections, bronchiectasis, and UTIs [20].

The range of antimicrobial agents deliverable via OPAT has expanded to reflect those available in the inpatient formulary [3]. In the UK, OPAT centres have administered a broad spectrum of IV antimicrobial agents, including beta-lactams (e.g., flucloxacillin), glycopeptides (e.g., teicoplanin), lipopeptides (e.g., daptomycin), oxazolidinones (e.g., linezolid), aminoglycosides (e.g., amikacin), tetracyclines (e.g., tigecycline), polymyxins (e.g., colistin), antifungals (e.g., caspofungin), antivirals (e.g., aciclovir), and antiprotozoals (e.g., sodium stibogluconate) [20]. Among these, ceftriaxone, teicoplanin, and ertapenem are the most frequently used, owing to their suitability for once-daily administration [20]. The selection of appropriate antimicrobial agent(s) for a particular infection is typically guided by local guidelines and protocols. The use of ‘last-resort’ reserve agents, such as colistin and tigecycline [21], is restricted and requires approval from a microbiologist or infectious diseases specialist.

Twenty-four-hour continuous infusion of piperacillin/tazobactam and flucloxacillin via elastomeric devices is now increasingly offered due to the availability of supporting antimicrobial stability data [22]. Newer long-acting agents, such as dalbavancin and rezafungin, which can be administered once weekly, are also being used in some services [23,24]. In addition, UK OPAT services are incorporating more complex oral antimicrobial therapies such as linezolid, alongside or instead of parenteral therapy [20,25,26].

### 2.2. National Initiatives

There is currently no national commissioning strategy or centralised programme for delivering, monitoring, and expanding OPAT services across the UK [27,28]. In the absence of a centralised OPAT programme, unlike in some European countries [28], the oversight and governance of OPAT services in the UK rest with individual hospitals and healthcare networks. As a result, there is considerable variation in OPAT practices across the country [4,5,9].

To promote and support OPAT services across the UK, BSAC established a national ‘OPAT Initiative’ in 2009. This Initiative has achieved significant milestones [29], including publishing Good Practice Recommendations (GPRs) for adult and paediatric OPAT programmes [4], creating an OPAT service directory, establishing national and regional networks, organising educational meetings at both national and regional levels, conducting antimicrobial stability tests, developing business case toolkits, and implementing a national outcomes registry system (NORS) to collect outcome reports for UK OPAT services [20].

The UK NORS, established in 2015, was closed in 2020. Despite being voluntary, it contained data from 57 OPAT centres, covering 27,841 patient episodes and 442,280 treatment days, with an average of 16 inpatient bed days saved per episode. The registry data provides a comprehensive overview of modern UK OPAT practice [20]. Its discontinuation, however, will make ongoing programmatic and national outcome assessments more challenging.

In 1998, a group of UK experts published a consensus statement on how OPAT programmes could be implemented in the country [30]. This statement has since been updated and replaced by GPRs, which incorporate new evidence to offer practical guidance on delivering OPAT in the UK and serve as a benchmark for service evaluation and quality improvement [31,32]. The most recent recommendations, published in 2019 by an expert working group on behalf of BSAC, cover various aspects of OPAT care, including team and service structure, patient selection and monitoring, antimicrobial delivery and management, outcome monitoring, and clinical governance [4]. These guidelines are currently under review, with updated recommendations expected later this year.

### 2.3. The Changing NHS Landscape

Established in 1948, the NHS has evolved into the world’s largest publicly funded healthcare system. It provides comprehensive and accessible healthcare to millions of UK residents, free at the point of use (or need), and is primarily funded through general taxation and National Insurance contributions, rather than through health insurance [33]. Since the late 1990s, healthcare in the UK has been a devolved matter, with each nation (NHS England, NHS Scotland, NHS Wales, and Health and Social Care Northern Ireland) responsible for the health needs of its respective populations.

Since its inception, the NHS has undergone various reforms to meet the changing needs of the population [33]. However, it now grapples with significant challenges, including funding constraints, staffing shortages, and rising demand. This demand is driven by an ageing population, the growing complexity of healthcare needs, and increasing public expectations, all of which have contributed to longer waiting times for treatment [34]. The COVID-19 pandemic further intensified the strain on the NHS [35]. A recent independent review of the NHS in England by Lord Darzi highlighted the severity of the crisis and outlined seven key areas for improvement, including improving productivity in hospitals and shifting more care to community settings [36]. This has prompted calls for radical reform, with some even describing the NHS as ‘broken’ [37,38]. In response, the UK government has launched the ‘Change NHS’ programme to gather public input for a new 10-Year Health Plan. This plan will prioritise three main ‘shifts’: moving care from hospitals to community settings, better use of technology, and emphasising disease prevention [8]. Furthermore, in March 2025, the UK government announced that NHS England, which manages the health service in England, would be dissolved within two years, with its responsibilities transferred back to the Department of Health and Social Care. The decision was justified as an effort to reduce bureaucracy and save money [39].

## 3. Benefits of OPAT Programmes

Table 1 outlines the advantages of OPAT. OPAT provides substantial benefits to patients and hospital systems, primarily by shortening inpatient stays (i.e., enabling early hospital discharge) and preventing hospital admissions. Several single-centre retrospective studies have demonstrated the benefits of OPAT for the NHS [10,40,41,42,43,44,45]. OPAT has been shown to be safe, clinically effective, cost-effective, and acceptable for treating a wide range of infections, with high levels of patient satisfaction [10,40,41,42,43,44,45]. Nevertheless, to the best of our knowledge, no randomised controlled trials (RCTs) have been conducted in the UK to directly compare OPAT with inpatient care [46]. As recognition of OPAT’s benefits continues to grow, conducting a definitive RCT to compare it with inpatient care is unlikely to be feasible and may pose ethical challenges.

### 3.1. Clinical Effectiveness and Safety

OPAT has consistently been shown to be both clinically effective and safe across various settings in the UK and worldwide [7,10,42,43,44,45]. Specific UK studies have highlighted its effectiveness and safety in treating infectious conditions such as endocarditis [47,48,49], cellulitis [50,51,52], and necrotising otitis externa [53], as well as for a range of antimicrobial agents [23,54,55]. For instance, Seaton et al. reported a success rate of 87% among 963 initial patient episodes of SSTIs managed by the Glasgow (Scotland) OPAT Service over a seven-year period [50]. Similarly, an analysis of the UK NORS (2015–2019) revealed a 92% positive infection outcome rate (cured or improved) across 27,841 treatment episodes [20].

A limited number of systematic reviews, encompassing both UK and predominately non-UK studies, have demonstrated that OPAT is comparable to inpatient care in terms of mortality, treatment failure, and adverse events [46,56,57,58]. For instance, Mitchell et al. examined the efficacy, safety, acceptability, and cost-effectiveness of OPAT in adult patients as part of a broader study exploring OPAT service delivery models in the NHS [56]. They found that OPAT achieved higher cure and improvement rates, with similar treatment durations, adverse drug reactions (ADRs), and mortality rates compared to inpatient care.

In general, comparative data on the clinical effectiveness and safety of OPAT suggest that transitioning clinically stable patients from hospital to OPAT is a viable and safe treatment pathway for those requiring parenteral antimicrobial therapy.

### 3.2. Cost-Effectiveness

Economic studies have demonstrated the cost savings associated with the delivery of OPAT within the NHS compared to inpatient care. For example, a review of the first ten years of the Sheffield (England) OPAT service found that the actual costs of establishing and running the service were approximately 39% of the equivalent national average for inpatient care [42]. Other studies have also demonstrated the cost-effectiveness of OPAT by examining the number of inpatient bed days saved [7,19,40,43]. For instance, Barr et al. reported savings of 39,095 inpatient bed-days across 2638 OPAT episodes over a ten-year period in Glasgow, Scotland [43]. Similarly, a review of the King’s College Hospital (London) OPAT service over a two-year period (2022–2024) treated 391 patients and saved 9516 hospital bed days, with estimated annual savings of £1–2 million based on inpatient costs of £300–600 per day [7].

The extent of savings varies depending on the OPAT care delivery model [59,60]. A decision-analytic modelling study, which used hospital records from England and published literature, estimated the cost per quality-adjusted life year gained from the NHS perspective. The study concluded that the ‘specialist nurse home visit’ model was the most cost-effective model for short-term treatments (up to seven days), while the ‘self-administration’ model was more cost-effective for longer treatments [60]. Another study, using a cost-minimisation analysis based on BSAC NORS, found that all OPAT service delivery models were consistently less expensive than equivalent inpatient stays in the NHS. Reported relative savings ranged from 13% to 56%, depending on the condition and the complexity of the OPAT model, including the early use of ‘complex’ highly bioavailable oral antimicrobial therapies [59].

The primary aim of OPAT is to deliver safe, high-quality, and cost-effective patient-centred care that is easily accessible. As no single approach suits all patients, UK OPAT services should offer a variety of care models to maximise benefits and meet the diverse needs of patients. Overall, OPAT represents a cost-effective use of NHS resources for treating various infections in patients who can be safely managed in a community setting.

### 3.3. Patient Experience and Acceptability

The preference of patients for OPAT over hospital care is well-documented [56,61,62]. Several qualitative studies have examined patients’ experiences and preferences regarding OPAT services in the UK [63,64,65,66]. One notable study by Twiddy et al. conducted 28 semi-structured interviews and a focus group to explore adult patients’ experiences receiving OPAT in four hospitals in Northern England [63]. Overall satisfaction with OPAT services was high, with patients identifying key advantages such as the comforts of home, avoidance of unnecessary hospitalisation, and minimal disruption to daily life, including work. Clear communication between the OPAT team and patients was highlighted as important.

Another study, conducted in Bristol, England, involved 92 patients and reported a mean overall satisfaction score of 9.6 out of 10. Patients noted that receiving treatment at home provided comfort, supported faster recovery, and reduced disruption to family life [65]. However, communication gaps between clinical teams and patients were also identified as an area for improvement. Other qualitative studies also have reported positive experiences with OPAT, including in a paediatric OPAT service [66].

### 3.4. Other Patient Benefits

Within the NHS, OPAT has been shown to improve patient-reported quality of life (QoL) measures and facilitate an earlier return to work or school for some individuals. For instance, a longitudinal pre-post study of 162 patients who received treatment through the Derby (UK) OPAT service between 2022 and 2023 reported significant improvements in health-related QoL scores. Of the 39 patients who were employed or in education prior to their illness, 18 (46%) were able to resume work or studies while receiving OPAT [67]. The ability to return to employment or maintain an income contributes to financial stability for many patients. The various OPAT delivery models offer treatment options that can be tailored to a patient’s individual needs, including their lifestyle, physical abilities, mental health needs, family situation, caregiving responsibilities, and financial resources. In addition, OPAT helps reduce costs by minimising the need for frequent hospital visits and associated transportation expenses.

Receiving treatment outside inpatient settings also addresses common challenges within hospitals, such as limited privacy, isolation from family and friends, and unfamiliar or potentially intimidating environments with consequential mental health impacts. Inpatient care can be particularly difficult for children, who are generally less adaptable to new surroundings and may have a limited understanding of their illness [3].

Evidence suggests that OPAT carries a lower risk of hospital-acquired infections, such as *Clostridioides difficile* infection (CDI) and *Staphylococcus aureus* bacteraemia [68]. Although there were initial concerns expressed about the use of broad-spectrum antibiotics like ceftriaxone or ertapenem in OPAT, UK studies have shown that the risk of CDI remains relatively low [69,70]. For example, a five-year study conducted in Sheffield, England, cross-referenced OPAT and hospital microbiology records to identify patients who received OPAT and subsequently developed CDI. The study reported a rate of six cases of CDI likely attributable to OPAT per 100,000 OPAT days. This is significantly lower than the national rate of 54 cases per 100,000 bed-days for hospitalised patients during the same period [70].

### 3.5. Organisational Benefits

OPAT optimises the efficient use of healthcare resources by easing inpatient staff workload and freeing up hospital beds [71,72]. This can allow hospitals to admit more patients or even reduce their bed capacity. It ensures that inpatient care is prioritised for those with the greatest need, while patients who are ambulatory and able to care for themselves or receive suitable home care are managed in a less resource-demanding environment. The significance of flexible and adaptable hospital capacity was especially evident during the COVID-19 pandemic.

Additionally, OPAT can streamline patient flow within the hospital. Patient flow, often a complex process, can involve multiple transitions—from primary care to emergency departments, admissions units, and finally, continuing care or specialist units. Each transition involves new clinical teams, the duplication of administrative tasks, and the potential for (communication) errors. Accessing OPAT before hospital admission (through direct referral from a primary care provider or self-referral) or from the emergency department can considerably simplify the process.

As most OPAT services in the UK are overseen by or involve local clinical microbiologists or infectious diseases (ID) physicians [5], OPAT ensures the consistent involvement of infection experts in patient care. This can improve adherence to care standards, enhance patient outcomes, and reduce inappropriate antimicrobial treatments [73,74]. By promoting appropriate antimicrobial use, minimising unnecessary hospital stays, and reducing the risk of hospital-acquired infections, OPAT plays a crucial role in preventing antimicrobial resistance (AMR).

## 4. Risks of OPAT

While OPAT provides significant benefits for both patients and hospital systems, its delivery is complex and carries inherent risks due to the reduced clinical monitoring and supervision compared to inpatient care. OPAT patients are susceptible to adverse events, including treatment failure and complications related to antimicrobial therapy, vascular access devices, underlying infections, pre-existing comorbidities (e.g., heart failure), and communication errors (e.g., incomplete referral information or inconsistent documentation of treatment plans across healthcare systems). These issues can lead to unplanned hospital readmissions.

Generally, at least 25% of patients receiving OPAT experience complications, which can range from mild antimicrobial side effects to more severe bloodstream infections associated with vascular access devices [4]. However, evidence suggests that the risk of treatment failure and adverse events is comparable to that observed during inpatient care [46,56,57,58]. Table 2 outlines the most commonly reported complications associated with OPAT. It is important to note that OPAT-related complications are inevitable for some patients due to the nature of the infections being treated, the potential toxicity of antimicrobials, the presence of vascular access devices, and the length of treatment [75]. To mitigate adverse events, careful patient selection is essential to ensure that only appropriate candidates who will genuinely benefit from OPAT receive it. Additionally, therapy should be administered for the shortest effective duration, with regular monitoring throughout the treatment course.

### 4.1. Overuse of OPAT

As OPAT services expand across the country, there is a risk of overuse of IV antimicrobial therapy in community settings [76]. The ease of access to OPAT may create an expectation for IV treatment, even for mild infections that could be treated with oral agents or no antimicrobial therapy at all [77]. Additionally, hospital capacity constraints in some regions may lead to pressure to treat more severely ill patients as outpatients, despite the potential benefits of inpatient care [77]. Furthermore, the logistic and staffing limitations associated with OPAT may drive the excessive use of broad-spectrum, once-daily parenteral antibiotics like ceftriaxone, rather than narrower-spectrum alternatives like benzylpenicillin, which require multiple daily doses but may be more appropriate [78]. There are also concerns that OPAT could shift some financial burdens of care from hospital systems to patients and their caregivers [77,79]. Several studies have shown that involving infection specialists in reviewing OPAT referrals reduces unnecessary use, enhances clinical care, and generates significant cost savings [80,81,82]. Thus, OPAT should be integrated into an antimicrobial stewardship programme, with infection specialist input to mitigate risks and enhance patient outcomes and sustainability.

### 4.2. OPAT-Related Complications

OPAT patients are at risk of complications related to the administered antimicrobials, intravascular access devices, and infusion devices (Table 2). Close monitoring, along with regular nursing and medical assessments, is essential to minimise risks and enable timely medical intervention [4]. It is crucial to inform patients about the potential adverse effects of antimicrobial therapy, complications associated with vascular access and infusion devices, the appropriate steps to take if issues arise, and emergency contact details. This proactive approach supports informed patient choice and engagement, helps facilitate timely intervention, and minimises the risk of severe outcomes.

#### 4.2.1. Adverse Drug Reactions

OPAT-related ADRs are common and can range from mild cutaneous rashes to more severe anaphylactic reactions. The risk of ADRs in OPAT patients ranges from 0% to 40%, depending on the definition of ADRs or the antimicrobial agent [83,84]. A review of the BSAC NORS (2015–2019) identified a median ADR rate of 1.9 per 1000 OPAT days, which was found to be higher in UK OPAT services treating a larger number of patients for shorter durations [20].

The risk of developing ADRs in OPAT often occurs during the first two weeks of treatment, frequently due to antibiotic reconciliation errors at hospital discharge [84]. The risks and types of ADRs in OPAT are likely comparable to those observed in the inpatient settings [15]. However, with longer durations of IV antimicrobial therapy, the risk of ADRs may increase [85,86]. For instance, severe neutropenia is a delayed complication of ceftriaxone, a commonly administered OPAT antimicrobial agent, and usually occurs after four weeks of treatment [87].

Minimising OPAT-related ADRs may require several strategies, including careful patient selection—taking into account comorbidities, renal function, and allergy history—appropriate antimicrobial choice, close monitoring through clinical assessments and laboratory investigations, and prompt management of any adverse events [4,84]. We suggest that future studies evaluate the actual impact of these interventions on reducing ADRs within the OPAT setting.

#### 4.2.2. Vascular Access-Related Complications

A safe and reliable vascular access device is crucial for the successful delivery of OPAT. In the UK, commonly used IV access devices for OPAT include peripheral venous catheters (for short-term treatment), midline catheters, and peripherally inserted central catheters (PICCs) (for longer treatments and vesicant antimicrobials). Other central vascular catheters, such as implanted ports and Hickman catheters, are also used [5].

These devices are prone to various complications, including thrombosis, central line-associated bloodstream infections, phlebitis, occlusion, and breakage (Table 2). Such complications, similar to those seen in inpatient settings [56], can disrupt antimicrobial therapy, lead to rehospitalisation, increase medical costs, and cause potentially fatal adverse events such as pulmonary embolism [88].

The incidence of catheter-related complications ranges from 0% to 29% [83], or 0 to 5 per 1000 OPAT days [89], depending on the complication type, with a higher risk associated with prolonged OPAT treatment [89,90,91]. Most reported complications are non-infectious, with line occlusion being the most common [89,92], while infection rates range from 0.2 to 2 per 1000 OPAT days [89,90,91,93]. The UK NORS (2015–2019) review identified a median complication rate of 1.4 per 1000 OPAT patient days, with device infections occurring at a rate of 0.3 per 1000 OPAT days [20]. Vascular device-related events were observed more frequently in services with longer mean OPAT episode durations.

Complication rates can vary depending on the catheter type. Midlines may have a lower infection risk but a higher risk of superficial vein thrombosis [94]. The risk of line-related thrombosis in OPAT is further influenced by the nature of the underlying infection and restricted mobility [95,96]. However, a recent systematic review suggests that the risk of catheter-related thrombosis in OPAT settings is lower than in critically ill and hospitalised patients [97] and does not support routine thromboprophylaxis or heparin flushes for preventing catheter-related thrombosis.

Overall, the use of intravascular access devices in OPAT settings results in acceptably low complication rates. However, careful consideration of modifiable risk factors, such as device type, insertion site, and techniques, along with appropriate line care, timely switching to oral therapy [88,98], and close monitoring, can reduce the risk of vascular access-related complications in OPAT.

#### 4.2.3. Infusion and Elastomeric Device Complications

Elastomeric infusion pumps are increasingly used in UK OPAT settings as they are easy to use, portable, and suitable for the continuous infusion of antimicrobials (subject to stability) with short half-lives, such as flucloxacillin and piperacillin/tazobactam, which require multiple daily dosing [22]. Elastomeric infusion pumps are non-electronic devices that contain a stretchable balloon reservoir, which contracts to provide a continuous flow at a preset rate, without relying on gravity or needles. While these devices offer the convenience of once-daily treatment and facilitate home-based care [99,100], complications can arise, resulting in treatment interruptions, inaccurate dosing, and reduced therapeutic efficacy.

Incomplete infusion, where the device fails to deliver the whole dose of antimicrobial medication within the prescribed time, is a common but often overlooked complication of elastomeric devices [22,101]. Incomplete infusion may occur due to improper use of the device (e.g., temperature, positioning, storage), a faulty device, or intravascular access-related issues [102,103,104]. Rarely, elastomeric devices may malfunction due to manufacturing defects or improper handling [102,104]. Issues like balloon rupture, leakage, or occlusion can interrupt the infusion process. Other complications include accidental disconnection, over-infusion, infection, air embolism, and allergic reactions.

Despite these challenges, elastomeric devices remain a valuable tool in OPAT, offering patients the benefit of easier and more comfortable self-administration and improved QoL [99,105]. They also permit the use of narrow-spectrum antimicrobial agents, such as flucloxacillin, which normally require multiple daily doses, thus contributing to AMS efforts within the OPAT setting. Regular monitoring, proper patient education, and adherence to best practices in device handling are essential to mitigate complications and ensure optimal OPAT outcomes.

#### 4.2.4. OPAT Failure

Like other medical interventions, OPAT poses a risk of treatment failure, which can negatively impact patients, increase medical costs, and contribute to AMR [106]. Several UK studies have examined the risk of treatment failure for different conditions [47,48,50,53,107,108], antimicrobial agents [23,54], and OPAT settings [109]. These studies have identified numerous factors associated with OPAT failure. For example, a 10-year review of a large West London (UK) OPAT service conducted in 2018 identified 69,610 days of treatment across 2870 patient episodes. It found that patients with successful outcomes had longer treatment courses (>14 days), did not experience adverse events, received treatment via peripheral vascular access, and were treated in an OPAT clinic rather than through self-administration [109].

Despite the risk of failure, evidence suggests that OPAT does not carry a higher risk of treatment failure compared to inpatient care [46,58]. Overall, pre-existing comorbidities and high Charlson comorbidity scores are the primary risk factors for OPAT failure [47,48,50,110]. Recognising these risk factors can help identify appropriate patients for OPAT and determine the safest mode of delivery.

#### 4.2.5. Patient Non-Compliance

Patient non-compliance can significantly influence the effectiveness and safety of OPAT, potentially leading to early treatment discontinuation, treatment failure, AMR, rehospitalisation, and increased medical costs. The reasons for non-compliance with OPAT are multifaceted [111]. Some patients may deliberately choose not to follow their prescribed treatment due to distrust in healthcare providers, personal beliefs, underlying mental health problems, or discomfort with aspects of the treatment, such as frequent administration and ADRs. Others may unintentionally fail to adhere due to cognitive impairments, misunderstanding of the treatment plan, or logistical difficulties, including transportation challenges [111]. Non-compliance is particularly prevalent among vulnerable populations, including people who inject drugs (PWID), the homeless, and those with underlying mental health issues [112]. Consequently, these groups are often excluded from OPAT treatment in the UK.

Enhancing compliance requires clear and comprehensive patient education about the treatment plan, potential adverse effects, and the importance of adherence. Involving family members or caregivers can provide additional practical and psychological support. Regular clinical follow-up, close monitoring, and addressing barriers to compliance—while tailoring the treatment plan to individual needs and preferences—are also crucial.

#### 4.2.6. Unplanned Hospital Readmission

Patient non-compliance, treatment failure, ADRs, vascular access-related complications, and non-OPAT-related adverse events (such as mechanical falls or new, unrelated infections, e.g., viral illness) can lead to unplanned readmission during OPAT treatment [75,113,114,115]. Reported hospital readmission rates vary widely, ranging from 1% to 26% [111]. However, the risk may be higher among vulnerable populations, such as PWID [116]. Worsening infection or a lack of response to treatment is the most frequently reported cause of readmission [75,113,114,115].

Several predictors of unplanned hospital readmission have been identified in the literature, particularly outside the UK.111 Predictive factors include complications related to vascular access devices and antimicrobial agents, patient- and infection-related factors, as well as factors related to OPAT service structure (Table 3). The most frequently reported predictor is the presence of comorbidities, such as underlying renal disease and malignancy [113,117].

Unplanned readmissions place a significant burden on both patients and the NHS, leading to increased healthcare costs. In the UK, a validated predictive model for 30-day unplanned hospital readmission was developed based on six easily obtainable variables: age, prior hospitalisation, Charlson comorbidity index score, treatment with multiple IV antimicrobial therapies, mode of OPAT delivery, and indication for OPAT [75,118]. Accurately predicting and preventing unplanned hospitalisations can enhance clinical outcomes by ensuring that OPAT is offered to the most suitable patients while identifying high-risk individuals who may benefit from more tailored care.

## 5. Barriers and Challenges

The successful implementation and expansion of OPAT programmes globally are hindered by various barriers and challenges that differ based on local healthcare system structure, regulatory frameworks, funding mechanisms, resource availability, sociocultural factors, and other contextual elements [111,119,120,121]. Similarly, the effective delivery of OPAT in different settings in the UK faces unique challenges. Addressing these issues will support OPAT growth and maximise its benefits for patients and the NHS.

### 5.1. Commissioning and Funding of Services

The NHS is grappling with deepening financial and operational challenges due to rising demand and chronic under-resourcing. This financial strain is affecting the provision of healthcare services, including OPAT programmes. The funding and commissioning of health services are complex due to multiple providers, varying systems, and competing priorities, and they have undergone frequent changes in recent years [33]. As healthcare is a devolved matter, each country within the UK (England, Scotland, Wales, and Northern Ireland) has its own separate system with distinct funding, policies, and priorities [33]. In England, most healthcare services are planned and arranged (commissioned) locally by integrated care boards (ICBs), which are overseen by NHS England. NHS England also directly commissioned certain specialised services, including some preventive public health services [33]. In contrast, healthcare services in Scotland, Wales, and Northern Ireland are primarily provided by NHS Scotland, NHS Wales, and Health and Social Care Northern Ireland, respectively. National health boards in each of these nations distribute funding to individual hospitals to meet the healthcare needs of their populations.

There is currently no national commissioning policy or dedicated funding mechanism for OPAT services in the UK. The reimbursement process for OPAT providers is highly variable and complex [122]. In some instances, OPAT may be perceived as an additional expense for already financially stretched hospitals rather than a cost-saving measure [59]. In other cases, OPAT services in the UK are set up and financed by acute hospital trusts, leveraging internal expertise to capitalise on the recognised benefits of OPAT, particularly in enhancing bed capacity and reducing overall costs for the organisation [122,123]. The inconsistent funding and commissioning arrangements pose major challenges to the broader development and expansion of OPAT services. To unlock the full potential of OPAT for the NHS and contribute to the government’s goal of providing healthcare services closer to people’s homes rather than in acute hospitals, as well as enabling equitable access to OPAT across the UK, it is crucial to address these commissioning and funding challenges.

Establishing a national commissioning mechanism, rather than relying on local negotiations with commissioners, could be a viable solution [6,9]. One proposal, suggested by Jones et al. [122], is the introduction of a standardised OPAT tariff to replace the existing array of funding arrangements. By setting the tariff at a rate that enables commissioners and OPAT providers to share cost savings resulting from OPAT, individual providers would receive explicit funding for their expertise (e.g., specialist pharmacist input) and the services they offer [123]. This approach would generate income proportionate to OPAT activity levels while also facilitating the planning and delivery of efficient services [122].

A national OPAT tariff would also grant commissioners the flexibility to commission the necessary expertise for designing and delivering safe OPAT care, whether through a single provider or a collaborative network of local providers [123]. In more rural areas, a hub-and-spoke model—similar to cancer care networks—could be adopted, where tertiary specialist centres, district hospitals, and community medical centres collaborate to deliver OPAT services [9].

### 5.2. Variation in Service Delivery

The expansion of OPAT services to dovetail with existing services and local needs has resulted in notable differences in OPAT clinical practice, governance, and adherence to the UK national OPAT guidelines [4,5,9]. For instance, a 2013 survey of 35 healthcare professionals providing OPAT in England found significant variations in both the scope of services offered and the models of care. Some respondents reported difficulties in maintaining or establishing OPAT services due to a lack of clear commissioning directives and/or limited engagement from senior managers [9]. Similarly, a 2017 survey of acute hospitals in the UK [5] reported that 39% (65) of the 165 health organisations that responded to the survey did not have an OPAT service, and 10% of OPAT services lacked an infection specialist, in contrast to the national OPAT guidelines [4], which recommend that OPAT MDT should include, at a minimum, an infection specialist.

Inconsistency in OPAT practice is not exclusive to the UK. Significant variations have also been observed in the US [124], Australia, and other European countries [125,126]. Deviations from national OPAT guidelines and differences in practice within the UK can be ascribed to several factors, including local guidelines and priorities, limited local funding and resources, institutional management, the availability of specialist expertise, gaps in knowledge, and geographical coverage [4,9,119,127]. For example, patients who live farther from hospitals are less likely to be offered home care by visiting nurses [9]. Well-established hospitals with specialised ID departments and clinical teams tend to have more robust OPAT programmes, supported by comprehensive local clinical guidelines and specialised nursing teams. These variations highlight areas for future OPAT research in the UK and present opportunities for targeted interventions.

While the impact of variations in OPAT practices on effectiveness and safety has not been extensively studied in the UK, standardising practice could offer significant benefits to both patients and hospital systems. These benefits include cost reduction, improved healthcare efficiency, better antimicrobial stewardship, and enhanced patient safety, resulting in better clinical outcomes and more sustainable care [128,129]. Encouraging collaboration among healthcare providers, such as establishing regional or geographical networks for OPAT services, could facilitate knowledge sharing, promote the adoption of best practices, and support both new and existing services. Eventually, this could lead to the standardisation of OPAT practice and the delivery of safer, more effective care across the UK. Additionally, introducing an OPAT service accreditation programme could improve adherence to the UK national OPAT GPRs, facilitating further improvements in patient safety and quality of care [130]. However, while striving for standardisation, it is also important to recognise the need to balance the benefits of standardisation with the advantages of allowing local interpretation and flexibility.

### 5.3. Inequitable Access to OPAT Care

The NHS’s goal is to provide equitable healthcare services. It aims to meet the needs of all individuals, regardless of their ability to pay. Despite the recent growth of OPAT in the UK, it is not yet widely available across all regions of the country. For instance, a 2021 report on OPAT in Scotland highlighted the absence of dedicated paediatric OPAT services in NHS Scotland and significant geographical variation in both access to and uptake of adult services [14].

Inequitable access to OPAT was clearly demonstrated in a study conducted within a large Scottish health board, which reviewed patients with cellulitis referred for OPAT care [131]. The study found that patients from the most deprived areas and women were significantly less likely to be referred. PWID, the homeless, and other hard-to-reach groups are often excluded from OPAT in the UK due to concerns about safety, adherence to treatment, and challenging social circumstances [132]. These variations contribute to the ‘postcode lottery’ effect and healthcare inequity, which may inadvertently exacerbate health disparities in the UK. As a result, some healthcare organisations miss out on the benefits of OPAT, and patients who could otherwise be treated at home instead occupy acute hospital beds due to a lack of local OPAT services. Similarly, the variable availability of OPAT services across the regions of the UK presents a significant challenge for patients who start OPAT in one region (e.g., at a specialist referral hospital) but need to continue their treatment in another (e.g., at their place of residence).

The ‘postcode lottery’ and inequity of access within the NHS extend beyond OPAT care [133,134]. Factors driving inequitable access to OPAT may include financial constraints, local policies, resource limitations (such as healthcare staff shortages), limited awareness of OPAT among commissioners, clinicians and healthcare providers, and patient-specific factors like transportation access, cultural and language barriers, and personal preferences [119,131]. Addressing these issues requires a multifaceted approach that tackles geographic, socioeconomic, cultural, and educational barriers. As OPAT care becomes more prevalent in the UK, it is vital that providers proactively address these disparities to prevent the worsening of existing inequalities. OPAT programmes should routinely assess the fairness of their services and actively implement strategies to mitigate any identified imbalances. Potential solutions include the use of telemedicine to support patients residing in remote areas [135], the provision of financial assistance and transportation support for those who require it, and the promotion of education and workforce training. Additionally, improved resource allocation, investment in infrastructure, and collaboration with other community-based care models (e.g., drug misuse services and virtual wards) could enhance OPAT services accessibility, thereby advancing the national goal of delivering more care closer to home.

### 5.4. Antimicrobial Stewardship (AMS)

Globally, AMS is increasingly recognised as a crucial component of healthcare. In the UK, OPAT has been recognised as one of the five options for ‘antimicrobial prescribing decisions’ outlined in NHS England’s Start Smart-Then Focus AMS guidance for hospitals. This strategy primarily aims to mitigate the risks associated with unnecessarily prolonged hospital stays [12]. While many institutions have well-established AMS programmes for inpatients, attention has only recently shifted to the outpatient setting. As OPAT use becomes more widespread, concerns about overuse and AMR have also grown [76,136].

The standard principles of good antimicrobial prescribing practice apply to OPAT, particularly the timely switch from IV to oral therapy (IVOST) [137]. However, key challenges in achieving effective AMS in OPAT settings include the unnecessary or prolonged use of IV antimicrobials when oral alternatives would be appropriate [137]. Ceftriaxone and ertapenem are commonly used antimicrobials in UK OPAT programmes. A review of the BSAC NORS (2015–2019) found that these two broad-spectrum agents were used in the treatment of over 55% of all adult patient episodes [20]. The widespread use of broad-spectrum, once-daily agents in OPAT—often chosen for convenience and logistical reasons—frequently comes at the expense of narrower-spectrum options, such as benzylpenicillin and temocillin, which require multiple daily doses [78,137]. This preference is further influenced by the limited availability of long-acting narrow-spectrum antimicrobial agents, insufficiently validated stability data for continuous infusion, and the need for frequent therapeutic drug monitoring (TDM) with certain narrow-spectrum agents, such as vancomycin [111]. Finally, clinical and operational factors, such as the frequency of patient monitoring and the composition of the OPAT team, have been found to impact the timely transition to oral therapy in a UK setting [52].

As OPAT continues to grow in the UK [4,5], it is essential that services be integrated into comprehensive infection and AMS programmes [4,137]. The selection of antimicrobials for OPAT should adhere to the local AMS policy. AMS principles in OPAT settings should focus on minimising unnecessarily prolonged IV therapy, using antimicrobials with the narrowest spectrum possible, promoting the transition from IV to oral treatment as soon as appropriate, and continually evaluating the need for source control. Every patient referred for OPAT should have a well-defined clinical and antimicrobial treatment plan that is regularly reviewed and adjusted as needed throughout the course of treatment. The involvement of infection specialists in OPAT programmes—particularly their role in preventing AMR by minimising unnecessary OPAT therapy and facilitating timely IVOST—cannot be overemphasised [81,82,138,139,140,141]. For instance, in a study that assessed the impact of an infection team review of patients receiving antibiotics in six UK hospitals, 89 patients were considered eligible for discharge to OPAT. Of these, 24 (27%) had their antibiotics stopped, and 55 (62%) were deemed suitable for oral outpatient treatment after the infection team review [138].

The availability of continuous infusion pumps and elastomeric devices has the potential to reduce the use of broad-spectrum antimicrobial agents in the OPAT setting, provided there is strong evidence supporting their stability. Portable continuous infusion pumps and elastomeric devices enable prolonged infusions of narrow-spectrum antimicrobials with time-dependent killing profiles and short half-lives, such as penicillin. This approach eliminates the need for multiple daily doses [105]. However, the limited availability of reliable stability data for many narrow-spectrum agents restricts their broader use in the OPAT setting [142].

Alongside other stewardship interventions, educating patients and healthcare professionals and fostering effective communication among all parties involved in patient care can significantly improve antimicrobial use [143]. The use of OPAT-specific AMS checklists, such as those proposed by Gilchrist et al., may also assist OPAT services in the UK in fulfilling their stewardship objectives [137].

### 5.5. Sustainability in OPAT

In general, the production, administration, and disposal of parenteral antimicrobials and their packaging have a significantly higher carbon footprint compared to oral agents [144]. The NHS is responsible for approximately 4–5% of the total UK carbon emissions [145]. The Government’s ‘Greener NHS’ campaign, launched in 2020, set a commitment for the NHS to become the world’s first net-zero carbon health service, as outlined in the Delivering a Net Zero NHS report [145]. The report provides a framework to help the NHS achieve net zero by 2040 for direct emissions and by 2045 for emissions it influences, a commitment later enshrined in the Health and Care Act 2022. As part of an expanding service within the NHS, OPAT practitioners have both the opportunity and responsibility to contribute to NHS decarbonisation.

Some aspects of OPAT services inherently offer more sustainable alternatives to traditional inpatient care models. These include reducing inpatient bed days, thus decreasing reliance on carbon-intensive infrastructure and clinical waste management [146], as well as supporting a speedier physical and mental recovery through home-based treatment [65,67]. However, other aspects may contribute to unnecessary carbon expenditure, such as excessive OPAT use or high rates of unplanned hospital readmissions. Among the different OPAT care models, self-administered OPAT has the lowest carbon footprint, followed by nurse-administered OPAT in the patient’s home, while daily travel to an OPAT clinic has the highest impact, as indicated by Cole et al. [147]. The authors assessed the environmental impact of these three OPAT care pathways in NHS England and estimated that, compared with inpatient care, self-administered, nurse-administered, and outpatient-administered OPAT were associated with 85%, 80%, and 71% CO_2_ reductions, respectively. Consequently, given the well-documented safety and efficacy of self-administered OPAT [10], as well as its lower cost compared to other models of OPAT care [59,60], we advocate that UK OPAT services should prioritise self- (or carer-) administration over other models for suitable patients.

Antimicrobial stewardship and sustainability often go hand in hand, as demonstrated by the benefits of a well-managed and timely IVOST. Thus, the need to optimise sustainable treatment choices further highlights the importance of regular review by the OPAT MDT, including infection specialists and antimicrobial pharmacists. Moving forward, OPAT service developers and clinical leaders must integrate sustainability considerations at all levels, from pathway design and procurement to day-to-day clinical decision-making.

## 6. Opportunities and Prospects

### 6.1. Future of OPAT in the UK

Due to the rising trend in antibiotic-resistant infections with no suitable oral treatment options in the UK [148], the number of patients eligible for OPAT care is expected to increase. Despite the challenges hindering the implementation of OPAT across the UK, its expansion and evolution at both local and national levels are anticipated, as seen in many other countries [4,11,111]. This growth will be fuelled by a growing body of evidence supporting its safety, cost-effectiveness, clinical efficacy, and patient preference [4,11,111]. Additionally, there is an increasing focus on shifting healthcare away from hospital settings [8], especially in the wake of the COVID-19 pandemic and the drive to reach net zero [145].

### 6.2. Opportunities for OPAT

The ongoing expansion of OPAT in the UK offers opportunities for innovative healthcare delivery methods and novel applications. It also has the potential to enhance efficiency, provide more integrated care (e.g., virtual wards) closer to patients’ homes, and maintain high-quality, sustainable healthcare within the NHS [6,149]. Evolving patient demographics, growing infection complexity, emerging evidence in infection management, advancements in medical care, and the development of new long-acting antimicrobial agents and delivery methods underscore the need for UK OPAT services to evolve, adopt new technologies, and extend beyond traditional practices [132]. Recent innovations and new applications of OPAT relevant to the NHS are explored below.

#### 6.2.1. Changes in OPAT Structure and Modes of Delivery

The landscape of UK OPAT is gradually evolving. In certain parts of the country, efforts to consolidate community services [150,151,152,153,154] have led to the integration of OPAT within a broader ‘Hospital in the Home’ model, similar to those in Australia [155] and Spain [156]. This expanded model supports the administration of complex medical therapies—such as parenteral nutrition, blood products, oxygen therapy, and IV fluids—in addition to parenteral antibiotic therapy, potentially opening the door to new cohorts of patients.

There is evidence that other infection-related services are increasingly being integrated into existing OPAT programmes in the UK. For instance, George et al. presented an innovative approach in South Wales, where long-acting injectable antiretroviral therapy for individuals with human immunodeficiency virus (HIV) was delivered through an OPAT service. This initiative received positive feedback from patients, OPAT nurses, and HIV clinicians [157]. In a US study, collaboration between an AMS Programme and an OPAT unit facilitated the rapid, safe, and effective delivery of monoclonal antibody therapy for mild-to-moderate COVID-19 infections [158].

It is anticipated that additional infection-related services, such as elective lumbar puncture (e.g., for patients with neurosyphilis) and penicillin allergy de-labelling programmes [159], will increasingly be delivered within OPAT services—particularly as the focus shifts toward oral antimicrobials over IV therapies. OPAT services should establish local protocols and algorithms to ensure the safe and effective delivery of such expanded services.

#### 6.2.2. Evolving Clinical Responsibilities

The roles of nurses and antimicrobial pharmacists in delivering OPAT in the UK are continuously evolving. For instance, a specialist nurse-led management approach for uncomplicated cellulitis has been described in Glasgow, demonstrating both safety and effectiveness without the need for hospital admission or regular medical staff input [160]. Similarly, studies outside the UK have shown that nurse- and pharmacist-led OPAT programmes are associated with reduced post-discharge costs, a lower risk of unplanned readmission and complications, and improved patient outcomes [161,162].

In the UK, advanced clinical practitioners, specialist nurses, and clinical pharmacists can obtain prescribing qualifications, expanding their roles in patient management and improving OPAT service efficiency. Within this framework, suitably qualified pharmacists and nurses can prescribe antimicrobials and other agents, provided they adhere to strict competency guidelines. This supports initiatives like complex outpatient antimicrobial therapy (COpAT) and reduces reliance on medical practitioners [132].

OPAT processes and patients are inherently complex, requiring an MDT to coordinate the care plans, including antimicrobial management, drug administration, and monitoring. Consequently, regardless of the specific team structure, the active involvement and oversight of a full OPAT team—including a lead nurse, antimicrobial pharmacist, and infection specialist—are crucial to achieving successful clinical outcomes [4].

#### 6.2.3. OPAT and Virtual Wards

There is a growing focus within NHS England on establishing virtual wards to provide coordinated healthcare [163], including oxygen therapy and physiological monitoring, delivered in patients’ homes. Traditionally offered in hospital settings, these services are now transitioning to home-based care.

**Figure 1:**
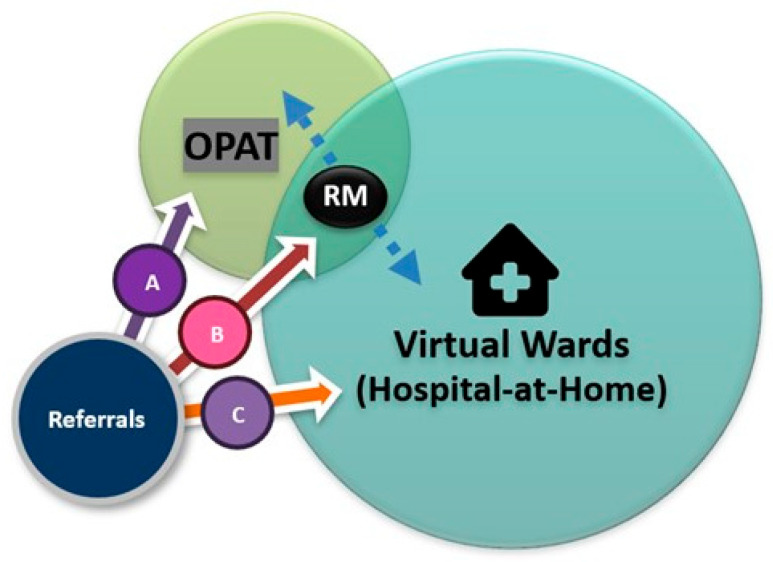
OPAT within the virtual wards concept. **Proposed Pathways: A**—Patients requiring only parenteral or complex oral antimicrobial therapy. **B**—Patients needing both acute care and parenteral or complex oral antimicrobial therapy. **C**—Patients suitable for acute clinical care outside the hospital setting, without requiring parenteral antimicrobial therapy. **RM** (Remote Monitoring)—collaborative care between the OPAT team and the virtual ward team. Patients may transition to either exclusive OPAT or virtual ward care upon completion of the required treatment (represented by the blue broken arrow).

Although distinct from virtual wards, OPAT aligns closely with the same NHS strategy of decentralising care, bringing treatment closer to home, and fostering a more integrated, community-based approach to healthcare provision [27]. Virtual ward care may include the administration of IV antibiotics, creating opportunities for OPAT involvement (Figure 1). OPAT teams play a crucial role in virtual wards by providing expert infection management, including input from antimicrobial pharmacists, and optimising antimicrobial therapies within the AMS framework. Similarly, the infrastructure of virtual wards is well-suited to support OPAT services, offering enhanced monitoring for selected patient groups [27]. This synergy fosters a more integrated and effective approach to home-based healthcare.

#### 6.2.4. Complex Outpatient Antimicrobial Therapy (COpAT)

The prolonged use of oral antimicrobial agents or therapies that require outpatient monitoring to optimise clinical outcomes and/or reduce the risk of adverse effects is referred to as COpAT [164,165]. The definition of complex antimicrobial therapy varies by patient and may encompass complex antimicrobial regimens, complex patients, or complex infections.

In recent years, emerging evidence suggests that oral antimicrobial agents can serve as effective alternatives to ‘traditional’ OPAT IV therapy for certain deep-seated infections, such as endocarditis [166], bone and joint infections (BJIs) [167], bloodstream infections [168], and intra-abdominal infections [169]. However, using oral therapies for these infections requires careful selection of antibiotics with good bioavailability and the ability to penetrate the site of infection. Moreover, careful monitoring is essential to evaluate drug interactions, ensure adherence, monitor ADRs, and track clinical progress [26]. Expert input from infection specialists and antimicrobial pharmacists is crucial and falls within the scope of OPAT care.

Interestingly, OPAT services in the UK have expanded to include the delivery of complex oral therapies in addition to IV therapy [20,132,170]. However, there is a lack of comprehensive studies in the UK evaluating COpAT. A study of two UK OPAT services revealed that patients preferred oral therapy over traditional OPAT or continued inpatient care [25].

Linezolid is being used more frequently in OPAT to facilitate an early switch from IV to oral treatment for BJIs [20,132]. However, close monitoring is necessary when linezolid is used for more than two weeks due to the risk of severe adverse events, particularly haematological toxicity [171]. Fluoroquinolones (e.g., ciprofloxacin, moxifloxacin, and levofloxacin) have also emerged as candidates for COpAT monitoring, especially following a safety update from the UK’s Medicines and Healthcare Products Regulatory Agency (MHRA). This update restricts the use of fluoroquinolone antibiotics due to their potential for serious musculoskeletal, cardiovascular, neurological, and psychiatric adverse effects, which can be disabling, long-lasting, and potentially irreversible [172]. Other oral antimicrobial agents suitable for COpAT include voriconazole, co-trimoxazole, chloramphenicol, and rifampicin-based regimens [132,165,170,173].

With an increasing emphasis on AMS and growing interest in the use of oral therapies, COpAT in the UK is anticipated to expand and evolve to encompass the management of more complex MDR infections [20,132,165]. To ensure optimal treatment and effective implementation of COpAT services, future studies should be designed to capture and evaluate this emerging area of practice.

#### 6.2.5. Long-Acting Parenteral Agents

A significant advancement in the approach to OPAT is the emergence of long-acting parenteral agents that can be administered at intervals longer than 24 h. For example, a novel IV antifungal, rezafungin, developed for invasive Candida infections, and two novel IV lipoglycopeptide antibiotics, dalbavancin and oritavancin, for Gram-positive infections, have extended elimination half-lives of approximately 5.5 days (133 h) [174], 14.5 days (346 h) [175], and 10 days (245 h) [176], respectively, allowing for a once-weekly dosing schedule.

Although originally approved for SSTIs, there is growing evidence supporting the effective use of dalbavancin and oritavancin in off-label situations, such as BJIs, bacteraemia, vascular graft infections, endocarditis, and spinal infections [176,177,178,179,180]. The extended dosing interval provided by these medications eliminates the need for an indwelling vascular access device and spares patients the inconvenience of daily dosing and frequent OPAT clinic visits. This is particularly beneficial for patients who face challenges in attending daily treatments (e.g., transportation constraints or living in remote locations), or for those who may not be suitable candidates for traditional OPAT due to concerns about compliance or the misuse of vascular access devices (e.g., PWID and patients with mental health conditions). Additionally, these medications can alleviate the burden on OPAT services with limited capacity by reducing the frequency of administration and the workload on nurses [4]. While these drugs currently have high acquisition costs, the overall treatment expenses may be offset by the savings from reduced OPAT visits, fewer vascular access complications, and averted or shortened inpatient stays [132].

Another area of interest is the dosing interval of traditional antimicrobial therapies, such as teicoplanin. Teicoplanin has been extensively used in UK OPAT services for the treatment of Gram-positive infections. Its extended elimination half-life makes it suitable for less frequent dosing. Studies have demonstrated the effectiveness of thrice-weekly teicoplanin in treating Gram-positive infections [181,182]. To support its administration two or three times a week in UK OPAT settings, dosing guidelines have been devised [183]. This approach has facilitated outpatient care for patients who cannot attend daily treatment or are unable to self-administer at home [43,50]. Nevertheless, the use of higher doses necessitates careful monitoring due to the potential for adverse events [47,50].

#### 6.2.6. OPAT in Hard-to-Reach Groups

In the UK, PWID, the homeless, and other hard-to-reach patient groups (e.g., those with mental health issues) are often deemed unsuitable for OPAT due to concerns about vascular access-related complications, the misuse of vascular devices, adherence to therapy, challenging social circumstances, patient and staff safety, and legal considerations [132,184]. However, these patient groups have been successfully treated with OPAT in other countries. For instance, a study in Singapore described the successful treatment of 29 PWID using a ‘package intervention’ that included strict patient selection, close monitoring, and preventive measures such as tamper-proof security seals over the vascular access device. There were no reported deaths or cases of vascular access misuse. However, six (21%) patients required hospital readmission due to infection- or treatment-related complications [185]. Similarly, a study conducted in the US demonstrated the effective use of OPAT in a closely monitored medical respite facility to treat infections in 53 homeless patients, many of whom were PWID. These patients were subject to a night curfew and had access to substance misuse services and opioid replacement therapy. Of these, 46 (87%) completed a defined course of antibiotic therapy and 34 (64%) were successfully treated with OPAT, resulting in an estimated cost saving of $25,000 per episode of care. However, 16 (30%) patients were readmitted during the study period. Of these, six (38%) admissions were not related to OPAT failure [186]. According to a literature review, OPAT can be considered safe and effective for PWID, as completion rates, mortality rates, and catheter-related complications were comparable to those observed in patients without a history of injecting drug use. While hospital readmission rates may be higher among PWID, instances of venous catheter misuse were low [184].

Careful patient selection is crucial to the success of OPAT among PWID and other hard-to-reach populations. OPAT treatment for these groups poses considerable challenges, necessitating a personalised model of care that incorporates an MDT approach. The model should include close clinical monitoring, access to substance misuse services and opioid replacement therapy [186], the optimisation of highly bioavailable oral therapies where possible, and the use of longer-acting parenteral antimicrobial agents to avoid the need for indwelling vascular access devices and reduce reliance on patient adherence to daily therapy [187].

#### 6.2.7. Continuous Antimicrobial Infusions and Infusion Devices

Administering narrow-spectrum beta-lactam antibiotics in OPAT often presents significant challenges Traditionally, OPAT favours once-daily or less frequent dosing for convenience and to minimise staffing demands. However, portable continuous infusion pumps, particularly elastomeric devices, offer a viable solution by enabling prolonged infusion of narrow-spectrum antimicrobial agents, such as flucloxacillin, which have a time-dependent killing effect and short half-lives. This approach eliminates the need for multiple daily dosing while supporting the principles of AMS [105].

Several UK studies have demonstrated the efficacy and safety of elastomeric devices in OPAT. For example, a five-year review of continuous infusion of flucloxacillin and piperacillin/tazobactam via elastomeric devices in the Derby OPAT service reported a high therapeutic success rate of 84%, with low complication and OPAT-related readmission rates [22]. Less frequent device changes—whether performed by healthcare professionals, patients, or caregivers—enhance patient-centred care, improve patient autonomy, alleviate the burden on healthcare systems, and reduce the need for nurse visits [100]. The use of elastomeric pumps in OPAT has also been associated with high patient satisfaction and acceptance [99], improved QoL [105], positive evaluations from nurses [188], and significant cost savings compared to conventional inpatient antimicrobial therapy [189]. In addition, the risk of line-related complications, such as infection and thrombosis, is minimised because the vascular device is accessed less frequently.

Elastomeric devices are more expensive than conventionally delivered OPAT antimicrobial therapies. However, acquisition costs may be reduced by preparing the elastomeric device in-house or adopting a ‘fresh fill’ approach, provided appropriate safeguards and risk assessments are in place [188,190]. Other drawbacks of elastomeric pumps include the absence of alarms or warnings in the event of occlusion, inconsistent infusion times, incomplete infusions, and restrictions on diluents [111].

Continuous infusion via an elastomeric device may not be appropriate for all antimicrobial agents, as their stability can vary over the infusion period, potentially reducing effectiveness and increasing the risk of toxic degradation products. The BSAC drug stability group has published data indicating that flucloxacillin [191], piperacillin/tazobactam [192], and temocillin [193] (reconstituted with citrate-buffered saline) are suitable for continuous infusions using elastomeric devices. However, meropenem is not recommended due to significant degradation within 24 h [194]. As OPAT continues to evolve in the UK, additional data on antimicrobial stability will be necessary to expand the range of agents that can be safely administered via extended infusions using elastomeric devices.

#### 6.2.8. Telemedicine in OPAT (Tele-OPAT)

With technological advancements, telemedicine has become an increasingly common method of delivering healthcare, particularly following the COVID-19 pandemic [195,196]. By leveraging digital tools, telemedicine fosters better communication between patients and healthcare providers, ensures continuity of care, improves accessibility and convenience, reduces travel costs and time, and facilitates more efficient use of resources [195,196].

OPAT guidelines recognise that telemedicine can support care delivery, especially in isolated and remote locations [4,15]. However, the adoption of telemedicine in OPAT (Tele-OPAT) has been relatively slow and limited, particularly in the UK [197]. Research on Tele-OPAT is limited, with most studies conducted in the US and Australia [198,199]. Evidence suggests that Tele-OPAT is cost-effective, associated with fewer unplanned readmissions, high patient satisfaction, and clinical outcomes and complication rates comparable to non-telemedicine OPAT care [135]. Additionally, it has the potential to reduce the carbon footprint of healthcare systems by minimising travel-related emissions and other environmental impacts.

As telemedicine gains popularity in healthcare, both new and established UK OPAT services should consider adopting it for care delivery, particularly for isolated populations, such as those in rural areas, nursing homes, or socioeconomically disadvantaged communities, in order to expand sustainable and equitable access to OPAT.

#### 6.2.9. Palliative OPAT

Historically, OPAT has been used to treat infections that follow a predictable course, respond well to antimicrobial therapy, and have a low likelihood of progression [200]. However, a small subset of patients with terminal illnesses or incurable infections require IV antimicrobial therapy despite being relatively stable and not needing inpatient care. ‘Palliative OPAT’ is an emerging concept referring to the use of OPAT for patients who need lifelong parenteral antimicrobials to manage, rather than cure, an infection (e.g., inoperable vascular graft infections) or to treat superadded infections in terminally ill patients (e.g., complicated intra-abdominal abscesses complicating metastatic cancer) [132].

Palliation is one of the aims of treatment outlined in the UK OPAT GPRs [4]. The use of OPAT for long-term infection suppression or palliation in incurable infections has been reported in a small number of UK OPAT services [201,202]. For example, a five-year retrospective review of the Nottingham OPAT service identified nine patients who received palliative OPAT [202]. This cohort accounted for 0.6% of OPAT patients but 8.6% of total bed days saved. Palliative OPAT provided positive outcomes for these patients and their families.

Although palliative OPAT can be complex and challenging, it helps preserve inpatient beds while enhancing QoL, providing symptom relief, improving well-being, and potentially prolonging life for patients with terminal infections [201,202]. With rising AMR, a growing population of complex patients, advancements in medical care, and a push for out-of-hospital care, the demand for palliative OPAT is expected to increase. This underscores the need for innovative approaches to effectively care for this patient group.

#### 6.2.10. Outpatient Subcutaneous Antimicrobial Therapy (OSCAT)

Administering antimicrobials via subcutaneous routes eliminates the need for a vascular access device, reducing associated risks such as infection and deep vein thrombosis. Subcutaneous antimicrobials have been widely used outside the UK, particularly in paediatric and geriatric care [203]. This approach offers convenience, especially for patients with poor venous access, while also reducing the burden on nurses [204]. Additionally, it can be performed in both home and outpatient settings.

Despite the lack of currently approved antimicrobials for subcutaneous administration, outpatient subcutaneous antimicrobial therapy (OSCAT) is widely used in France and other European countries [204,205]. Antimicrobial agents with extended half-lives, complete absorption, favourable pharmacokinetic/pharmacodynamic (PK/PD) profiles, and tolerable local adverse event rates (e.g., pain) are considered ideal for OSCAT [204]. According to a large French national survey of ID physicians and geriatric practitioners, ceftriaxone is the most prescribed subcutaneous antibiotic, followed by teicoplanin and ertapenem [205]. Studies have shown that the PK/PD profiles and clinical effectiveness of ceftriaxone, teicoplanin, and ertapenem are comparable when administered via the subcutaneous or IV routes [206]. Other antibiotics that have been administered subcutaneously include aminoglycosides, amoxicillin, amoxicillin-clavulanic acid, cefepime, ceftazidime, fosfomycin, imipenem, and piperacillin/tazobactam [205]. Typical adverse events associated with subcutaneous antimicrobials include pain, hematoma, induration, and erythema at the site of injection [204,205]. However, these effects are generally mild and transient. Severe local complications, such as skin necrosis, are rare and have been reported more frequently with subcutaneous aminoglycosides [204].

Advancements in wearable, on-body subcutaneous delivery systems are further enhancing the feasibility of OSCAT by promoting patient autonomy, minimising the risk of line-related complications, and potentially reducing the need for direct healthcare professional supervision [204]. This innovative approach is particularly beneficial for patients with poor venous access, those at risk of misusing vascular devices (e.g., PWID), and individuals who may struggle to maintain a venous access device (e.g., those with cognitive or behavioural disorders). However, further research is needed to fully assess the practicality and clinical effectiveness of OSCAT in UK OPAT settings.

## 7. Conclusions

This review highlights that OPAT represents a safe, clinically effective, and cost-efficient use of NHS resources. Although OPAT carries inherent risks, its safety profile supports its use in appropriately selected patients, provided that comprehensive systems are in place to mitigate potential complications. Nevertheless, its implementation across the country remains patchy and is not without challenges. While the NHS may be viewed as ‘broken,’ ongoing reforms suggest that OPAT will play an increasingly vital role in healthcare delivery. To unlock OPAT’s full benefits, a coordinated strategy is necessary. Supported by high-quality, ongoing research, this strategy should encompass innovative treatment approaches, broader clinical applications, the creation of a standardised funding model, and a national commissioning policy. Ultimately, OPAT in the UK should transition from a ‘nice-to-have’ option to a standard-of-care ‘must-have’ service wherever there is a clinical need [29].

## Figures and Tables

**Table 1:** Patient and organisational benefits of OPAT.

Patient Benefits	Organisational Benefits
Offers treatment tailored to individual needs	Provides significant cost savings
Enhances patient convenience and privacy	Improves efficient use of inpatient resources
Minimises disruption to family life	Improves inpatient capacity
Reduces the time and cost incurred by families visiting patients in the hospital	Enhances sustainability savings and lowers carbon footprint
Provides greater freedom of movement	Relieves congestion in emergency departments
Improves quality of life and overall wellbeing	Enhances patient flow
Improves patient experience and satisfaction	Enables bed reduction and reconfiguration
Increases the likelihood of an earlier return to work or school	Reduces hospital staff workload Enhances opportunities for antimicrobial stewardship
Reduces social and psychological stress Reduces risk of hospital-acquired infections	Ensures consistent input from infection specialist in patient care

**Table 2:** OPAT-related complications.

Vascular Access Complications	Drug-Related Complications	Infusion Device Related	Others
Allergy to dressing	Antibiotic-associated diarrhoea	Battery failure (electronic pumps)	Communication errors
Breakage	Blood dyscrasia	Device leakage	Non-adherence
Dislodgement	*Clostridioides difficile* infection	Device malfunction	Readmission
Infection	Electrolyte disturbances	Disconnection or accidental removal	Treatment failure
Migration	Gastrointestinal effects	Flow rate irregularities	
Occlusion	Hepatotoxicity	Incomplete infusion	
Thrombophlebitis	Nephrotoxicity		
Thrombosis	Neurotoxicity		
	Rash		

**Table 3:** Common predictors of unplanned hospital readmission during OPAT.

Patient-Related	Infection-Related	Antimicrobial- Related	Vascular Access-Related	OPAT Structure
Existing comorbidities (e.g., chronic renal disease, malignancy) High CCI Score	Condition treated (e.g., endovascular, prosthetic infection)	Adverse drug events Antimicrobial agent (e.g., glycopeptides, aminoglycosides)	Infection Line patency issue Thrombosis	Inadequate OPAT follow-up Mode of OPAT delivery (e.g., skilled nursing facility, subacute rehabilitation centre)
Older age Prior hospital admission Substance misuse	Multidrug-resistant Organisms Prolonged therapy	Concurrent IV therapy		Non-availability of test results

CCI, Charlson Comorbidity Index; IV, intravenous; OPAT, outpatient parenteral antimicrobial therapy.

## Data Availability

No new data were created or analysed in this study. Data sharing is not applicable to this article.

## Abbreviations

The following abbreviations are used in this manuscript:

ADRs	Adverse drug reactions
AMR	Antimicrobial resistance
AMS	Antimicrobial stewardship
BJIs	Bone and joint infections
BSAC	British Society for Antimicrobial Chemotherapy
CCI	Charlson comorbidity index
CDI	*Clostridioides difficile* infection
COpAT	Complex outpatient antimicrobial therapy
GPRs	Good practice recommendations
HIV	Human immunodeficiency virus
ICB	Integrated care board
ID	Infectious diseases
IV	Intravenous
IVOST	Intravenous to oral therapy
MDR	Multi-drug resistant
MDT	Multidisciplinary team
MHRA	Medicines and Healthcare Products Regulatory Agency
NHS	National Health Service
NORS	National outcomes registry system
OPAT	Outpatient parenteral antimicrobial therapy
OSCAT	Outpatient subcutaneous antimicrobial therapy
PICCs	Peripheral inserted central catheters
PK/PD	Pharmacokinetic/pharmacodynamic
PWID	People who inject drugs
RM	Remote monitoring
SSTIs	Skin and soft tissue infections
QoL	Quality of life
RCTs	Randomised controlled trials
TDM	Therapeutic drug monitoring
Tele-OPAT	Telemedicine in OPAT
UK	United Kingdom
US	United States
UTIs	Urinary tract infections
